# Integrative Proteomic and MicroRNA Analysis: Insights Into Mechanisms of Eyestalk Ablation-Induced Ovarian Maturation in the Swimming Crab *Portunus trituberculatus*

**DOI:** 10.3389/fendo.2020.00533

**Published:** 2020-08-14

**Authors:** Xianliang Meng, Mengqian Zhang, Baoquan Gao, Jianjian Lv, Jian Li, Ping Liu

**Affiliations:** ^1^Key Laboratory of Aquatic Genomics, Ministry of Agriculture and Rural Affairs, Yellow Sea Fisheries Research Institute, Chinese Academy of Fishery Sciences, Qingdao, China; ^2^Laboratory for Marine Fisheries Science and Food Production Processes, Qingdao National Laboratory for Marine Science and Technology, Qingdao, China; ^3^Key Laboratory of Sustainable Development of Marine Fisheries, Ministry of Agriculture and Rural Affairs, Yellow Sea Fisheries Research Institute, Chinese Academy of Fishery Sciences, Qingdao, China; ^4^College of Marine Life and Fisheries, Jiangsu Ocean University, Lianyungang, China

**Keywords:** crab, eyestalk ablation, miRNA, proteome, ovarian development

## Abstract

Eyestalk ablation is the most common method to induce ovarian maturation in decapod crustacean aquaculture, but it jeopardizes broodstock survival and larvae production. It is important to understand the molecular basis underlying the maturation triggered by ablation and thereby develop an alternative measure for maturation manipulation. In this study, we investigate alterations of ovarian proteome and miRNA profile after ablation in a commercially important marine crab *Portunus trituberculatus*. Quantitative proteomic analysis using iTRAQ reveals that 163 proteins are differentially expressed following ablation, and modulation of methyl farnesoate metabolism and activation of calcium signaling may play important roles in the ovarian maturation induced by ablation. miRNA expression profiling identifies 31 miRNAs that show statistically significant changes. Integration of miRNA and proteome expression data with miRNA target prediction algorithms generates a potential regulatory network consisting of 26 miRNAs and 30 proteins linked by 71 possible functional associations. The miRNA-protein network analysis suggests that miRNAs are involved in promoting ovarian maturation by controlling expression of proteins related to methyl farnesoate synthesis, calcium signals, and energy metabolism. Experimental validation and temporal expression analysis indicate multiple miRNAs can act synergistically to regulate expression of Farnesoic acid O-methyltransferase and Calmodulin. Our findings provide new insights for elucidating the mechanisms underlying eyestalk ablation-induced ovarian maturation and could be useful for devising an alternative technique for manipulating reproduction in *P. trituberculatus* and other decapods.

## Introduction

The eyestalk, where the X-organ/sinus gland (XO/SG) complex is located, is an important neuroendocrine system in crustaceans, which is the major site of production and storage for a variety of important neuropeptides, such as the gonad-inhibiting hormone (GIH), the molt-inhibiting hormone (MIH), the pigment-dispersing hormone (PDH), and the red pigment-concentrating hormone (RPCH) (1, 2). Among these neuropeptides, GIH is considered to play a key role in regulating female crustacean reproduction by repressing ovarian maturation and spawning (3). In decapod aquaculture practice, removal of GIH by eyestalk ablation (ESA) has been the most common method to induce ovarian maturation (4). However, in addition to GIH, secretion of other neuropeptides from the eyestalk is also impaired by this method, which results in significant hormonal imbalance and undesired impacts on not only the survival of broodstock, but also the quantity and quality of larvae produced (5). Hence, an alternative technique for maturation manipulation without ESA has been a long-term goal for the decapod culture industry.

To develop new techniques for triggering maturation, it is important to understand the molecular mechanisms of ablation-induced ovarian maturation. During the past decade, many efforts have been made to elucidate the molecular effects of ESA on ovarian development in decapods (6–10). With high-throughput transcriptome analysis, recent research provides a global picture of gene expression changes at the transcriptional level after ablation. Uawisetwathana et al. (11) find that eyestalk ablation activates several signal pathways important for ovarian development, such as the gonadotropin-releasing hormone (GnRH) signaling pathway and the progesterone-mediated oocyte maturation pathway, which promotes ovarian maturation in *Penaeus monodon*. Lee et al. (12) report that ESA can modulate gene expression in the hepatopancreas of *Litopenaeus vannamei*, upregulating genes related to metabolic processes as well as those associated with immunity and stress responses. These studies expand our knowledge on the transcriptional regulation that mediates ESA-induced maturation. Nevertheless, transcription change does not depict the changes of protein levels with complete accuracy because gene expression can also be modulated at the post-transcriptional and translational levels. To date, no information is available regarding gene expression regulation at post-transcriptional and translational levels following ESA.

MicroRNAs (miRNAs), a group of small non-coding RNAs, function as key post-transcriptional regulators of gene expression by complementary binding to 3′ untranslated regions of their target mRNA, leading to mRNA destabilization, or protein translation blockage (13, 14). They are involved in many fundamental biological processes, such as development, metabolism, cell proliferation, and signal transduction (15–17). Recent evidence has revealed that miRNAs are critical in ovarian development and maturation in decapod crustaceans. In *Eriocheir sinensis*, miR-2, and miR-133 are demonstrated to regulate meiotic oocyte maturation by modulating cyclin B expression (18). A number of miRNAs have been found to exhibit ovary-biased expression in *L. vannamei, Macrobrachium nipponense, Portunus trituberculatus*, and *Scylla paramamosain*, and many of the predicted target genes for these miRNAs are crucial in regulating ovarian development, indicating the important functions of miRNA in ovarian development (19–22). However, the roles of miRNA in ESA-induced ovarian maturation is still unknown.

Proteomic analysis is a tool that facilitates research on comprehensive protein expression profiling and identification of individual proteins involved in specific biological responses. It has been widely utilized in studying reproductive development in mammals, fish, and insects (23–25). In crustaceans, only a few proteomic studies have been conducted on ovarian development, and these studies all rely upon 2-D gel electrophoresis data (26–28), which cannot identify low abundant proteins, proteins with low or high molecular weights, and proteins that are excessively acidic or basic as well as hydrophobic proteins (29, 30). The isobaric tags for relative and absolute quantitation (iTRAQ) is a mass spectrometry–based proteomics technique established in recent years and has overcome some of the drawbacks of 2-D gel electrophoresis; it is sensitive and can detect large proteins and low-abundant proteins with high throughput and low experimental error (31). Hence, iTRAQ should be an ideal approach to study protein expression changes after ESA.

The swimming crab *P. trituberculatus* (Crustacea: Decapoda: Brachyura) is an important fishery and aquaculture species, widely distributed in the estuary and coastal waters of Korea, Japan, China, and Southeast Asia (32). This species supports a large aquaculture industry in China, and its farming developed quickly during the last decade. In 2017, the production of *P. trituberculatus* reached 119,777 t (China Fishery Statistical Yearbook, 2018). However, due to rapid expansion of crab farming and lack of an effective technique for reproductive manipulation, high-quality seed production cannot fulfill the demand, which restricts further development of the industry (33, 34). Therefore, it is crucial to understand the regulatory mechanisms of ovarian maturation and improve the means of artificial seed production. In this study, we investigate the alteration in proteome and miRNA transcriptome following ESA. The results can improve our understanding of molecular mechanisms underlying ESA-induced ovarian maturation and provide useful information for developing new reproduction manipulation techniques.

## Results

### Protein Expression Altered by ESA

To identify proteins involved in ESA-induced ovarian maturation, iTRAQ was employed to assess ovarian protein expression changes at the fourth day after ESA. In total, 483,601 spectra were generated from the ovary of the eyestalk-intact (ESI) and ESA crabs. Based on the spectral data, 15,377 peptides and 3400 proteins were identified (cutoff: Mascot Percolator *q*-value <= 0.01). With the criteria of fold change ≥1.2 and *p* < 0.05, 163 proteins were identified to be differentially expressed (DE) between ESI and ESA crabs; a great majority (132 proteins, 80.98%) of the DE proteins showed upregulation in ESA individuals (Table 1).

**Table 1:** List of the annotated proteins that exhibited differentially expression after ESA.

	**NCBI Accession**	**Protein name**	**Fold change**
1	XP_003702718.1	Peritrophin-1-like	3.66
2	XP_012151113.1	Fibrillin-2-like isoform X2	3.08
3	AAY56093.1	Death-associated protein-like	2.32
4	ACZ02405.1	Heat shock protein 70	2.28
5	XP_003248548.1	Clathrin light chain-like	1.95
6	XP_002431005.1	Ubiquitin-fold modifier 1 precursor	1.90
7	XP_002404909.1	Prefoldin	1.84
8	AFE88627.1	Thioredoxin 2	1.79
9	KDR08860.1	Tumor protein D54	1.79
10	XP_004922887.1	BolA-like protein	1.76
11	XP_012176731.1	Fibril-forming collagen alpha chain like	1.76
12	XP_974307.1	Similar to par-6 gamma	1.74
13	ADZ96217.1	JHE-like carboxylesterase 1	1.70
14	EFN74540.1	Sorting nexin-12	1.70
15	AFS60116.1	Selenoprotein M	1.69
16	NP_001037686.1	Aspartylglucosaminidase	1.67
17	BAJ22990.1	Cytochrome c	1.67
18	ACL26692.1	Farnesoic acid O-methyltransferase	1.67
19	XP_001865898.1	Antioxidant enzyme	1.66
20	AAY57406.1	Program cell death 5-like	1.66
21	ACU82846.1	Acyl-CoA-binding protein	1.64
22	ACO11851.1	RNA-binding protein 1	1.64
23	AAO73307.1	Ovary development-related protein	1.63
24	ACO11926.1	Charged multivesicular body protein 5	1.61
25	ACJ53746.1	Peroxiredoxin 6	1.61
26	XP_001942794.1	39S ribosomal protein L12, mitochondrial-like isoform 1	1.60
27	ACL13568.1	AMP-activated protein kinase alpha subunit	1.60
28	ACY66390.1	FK506-binding protein 1A	1.60
29	ABI98678.1	Ubiquitin-conjugating enzyme	1.59
30	XP_972770.2	Insulin receptor	1.58
31	ADE60733.1	Myosin essential light chain	1.57
32	NP_001103783.1	Tropomyosin-2 isoform 3	1.57
33	ABF55966.2	Cleavage stimulation factor 64-kDa subunit	1.56
34	EFN66390.1	PERQ amino acid-rich with GYF domain-containing protein 2	1.56
35	XP_796085.2	Transcription and mRNA export factor ENY2	1.56
36	XP_011300302.1	Prefoldin subunit 1	1.55
37	KDR23647.1	Protein phosphatase inhibitor 2	1.55
38	ACR56783.1	Small ubiquitin-like modifier-1	1.55
39	ACY66642.1	Thymosin beta	1.55
40	EGI57685.1	Non-specific lipid-transfer protein	1.54
41	KDR12501.1	Spondin-1	1.54
42	EFN86015.1	GS1-like protein	1.53
43	XP_003723328.1	ES1 protein homolog, mitochondrial-like	1.52
44	XP_011501070.1	Far upstream element-binding protein 1 isoform X3	1.52
45	XP_003445744.1	Methylmalonyl-CoA epimerase	1.52
46	EGW07359.1	Peptidyl-prolyl cis-trans isomerase, mitochondrial	1.52
47	XP_008470922.1	Verprolin-like	1.52
48	XP_003451145.1	Aminopeptidase W07G4.4-like	1.51
49	XP_012259502.1	DnaJ homolog subfamily B member 11	1.51
50	XP_001847373.1	Ubiquinol-cytochrome c reductase complex 14 kDa protein	1.51
51	ADW24146.1	Vesicle-associated membrane protein-associated protein	1.5
52	AAX94762.1	Vitellogenin	1.5
53	XP_003707900.1	28S ribosomal protein S36, mitochondrial-like	1.49
54	XP_002427853.1	Protein-L-isoaspartate O-methyltransferase	1.48
55	BAM18170.1	Prefoldin subunit	1.47
56	AGF39576.1	Double WAP domain-containing protein	1.46
57	XP_002413321.1	LIM domain-binding protein	1.46
58	XP_001660469.1	Low-density lipoprotein receptor	1.46
59	EGI66356.1	Prefoldin subunit 2	1.46
60	ELW71144.1	Calmodulin	1.45
61	KDR23803.1	Outer dense fiber protein 3	1.45
62	XP_001947263.2	Upstream activation factor subunit spp27-like	1.45
63	XP_008473520.1	Pumilio homolog 1-like	1.44
64	NP_001090127.1	Tubulin folding cofactor B	1.44
65	ACO36738.1	Ubiquitin carboxyl-terminal esterase L3	1.43
66	KMQ90936.1	Barrier-to-autointegration factor	1.42
67	NP_001156264.1	Calcium-regulated heat stable protein 1	1.42
68	XP_002423140.1	Charged multivesicular body protein 4C	1.42
69	XP_003690694.1	Synaptosomal-associated protein 29-like	1.42
70	ACI13851.1	Extracellular copper-zinc superoxide dismutase	1.41
71	XP_002425615.1	Methionyl-tRNA synthetase	1.41
72	XP_008199673.1	Nuclear protein MDM1 isoform X4	1.41
73	XP_003486756.1	Stress-induced-phosphoprotein 1-like isoform 1	1.41
74	ETN61219.1	Aldehyde oxidase	1.40
75	ADO00931.1	Calnexin	1.40
76	XP_002413938.1	Low-density lipoprotein receptor	1.40
77	KDR20387.1	Phosphoglycerate mutase 2	1.40
78	ACY66501.1	60S acidic ribosomal protein P2	1.39
79	XP_004065994.1	Cytosol aminopeptidase-like	1.39
80	XP_011416416.1	Histidine triad nucleotide-binding protein 2	1.39
81	EKC27215.1	Ras GTPase-activating protein-binding protein 2	1.39
82	EKC33829.1	Methenyltetrahydrofolate synthetase domain-containing protein	1.38
83	XP_011157802.1	Short-chain specific acyl-CoA dehydrogenase	1.38
84	XP_012150508.1	Multiple coagulation factor deficiency protein 2 homolog	1.37
85	XP_002409815.1	PDZ domain-containing protein	1.37
86	XP_001943654.1	Bifunctional methylenetetrahydrofolate dehydrogenase/cyclohydrolase	1.36
87	NP_001164152.1	Held out wings	1.36
88	ACR54112.1	Ribosomal protein P1	1.36
89	KDR12892.1	Syntaxin-12	1.36
90	XP_003400244.1	Cyclin-dependent kinase 6-like	1.35
91	XP_005175277.1	ATP synthase subunit d	1.34
92	ACH88358.1	Cell division cycle 2	1.34
93	ADN52396.1	Triosephosphate isomerase	1.34
94	EFA07536.1	RAE1 RNA export 1 homolog	1.33
95	ACO14747.1	Calponin-3	1.32
96	NP_001020355.1	DnaJ homolog subfamily B member 9 precursor	1.32
97	ACZ06791.1	Eukaryotic translation initiation factor 5A	1.32
98	AET36895.1	Peroxiredoxin 2	1.32
99	AAC78141.1	Phosphopyruvate hydratase	1.32
100	AFC17961.1	O-methyltransferase	1.31
101	XP_002415663.1	Alternative splicing factor ASF/SF2	1.30
102	EKC42097.1	Cathepsin F	1.28
103	ACI46952.1	Cyclin B	1.28
104	KDR07772.1	Plastin-2	1.28
105	XP_012267954.1	Golgi resident protein GCP60	1.27
106	XP_972648.1	Similar to adaptin ear-binding coat-associated protein 2	1.25
107	ACY66506.1	Ubiquitin associated protein 2-like protein	1.25
108	KFM60612.1	DnaJ-like protein subfamily A member 2	1.24
109	XP_003705474.1	Aconitate hydratase	1.23
110	ADQ55791.1	Antimicrobial peptide hyastatin	1.23
111	CAA72032.2	Masquerade-like protein	1.22
112	XP_973346.1	Phosphoacetylglucosamine mutase	0.83
113	AAC64660.1	Pacifastin heavy chain precursor	0.82
114	AEF32710.1	Translationally controlled tumor protein	0.81
115	ACY66537.1	60S ribosomal protein L27	0.80
116	AAZ22828.1	Lymphoid organ expressed yellow head virus receptor protein	0.80
117	KFM60603.1	60S ribosomal protein L7a	0.79
118	ABQ10738.1	Cathepsin D	0.79
119	XP_011136175.1	Proteasome subunit alpha type-5	0.79
120	XP_972566.1	Succinate semialdehyde dehydrogenase, mitochondrial	0.79
121	AET34923.1	Peroxiredoxin 1	0.78
122	XP_002401133.1	Ribosomal protein S26	0.78
123	NP_001037263.1	Ribosomal protein S8	0.77
124	XP_003705948.1	1,2-dihydroxy-3-keto-5-methylthiopentene dioxygenase-like	0.75
125	XP_002423307.1	cAMP-dependent protein kinase catalytic subunit	0.73
126	ACN87221.1	Phenoloxidase activating factor	0.72
127	XP_007442568.1	Glutathione peroxidase 7-like	0.71
128	KFM75426.1	Protein canopy-like protein	0.71
129	ABX71209.1	Glycosyl-phosphatidylinositol-linked carbonic anhydrase	0.69
130	AAW57889.1	Hemocyanin subunit 1	0.69
131	AAW57890.1	Hemocyanin subunit 2	0.69
132	AAW57891.1	Hemocyanin subunit 3	0.65
133	AAA96966.2	Hemocyanin subunit 6	0.59
134	AAF64305.1	Hemocyanin subunit	0.56
135	EGI63299.1	Histone-lysine N-methyltransferase trr	0.24

**Figure 1:**
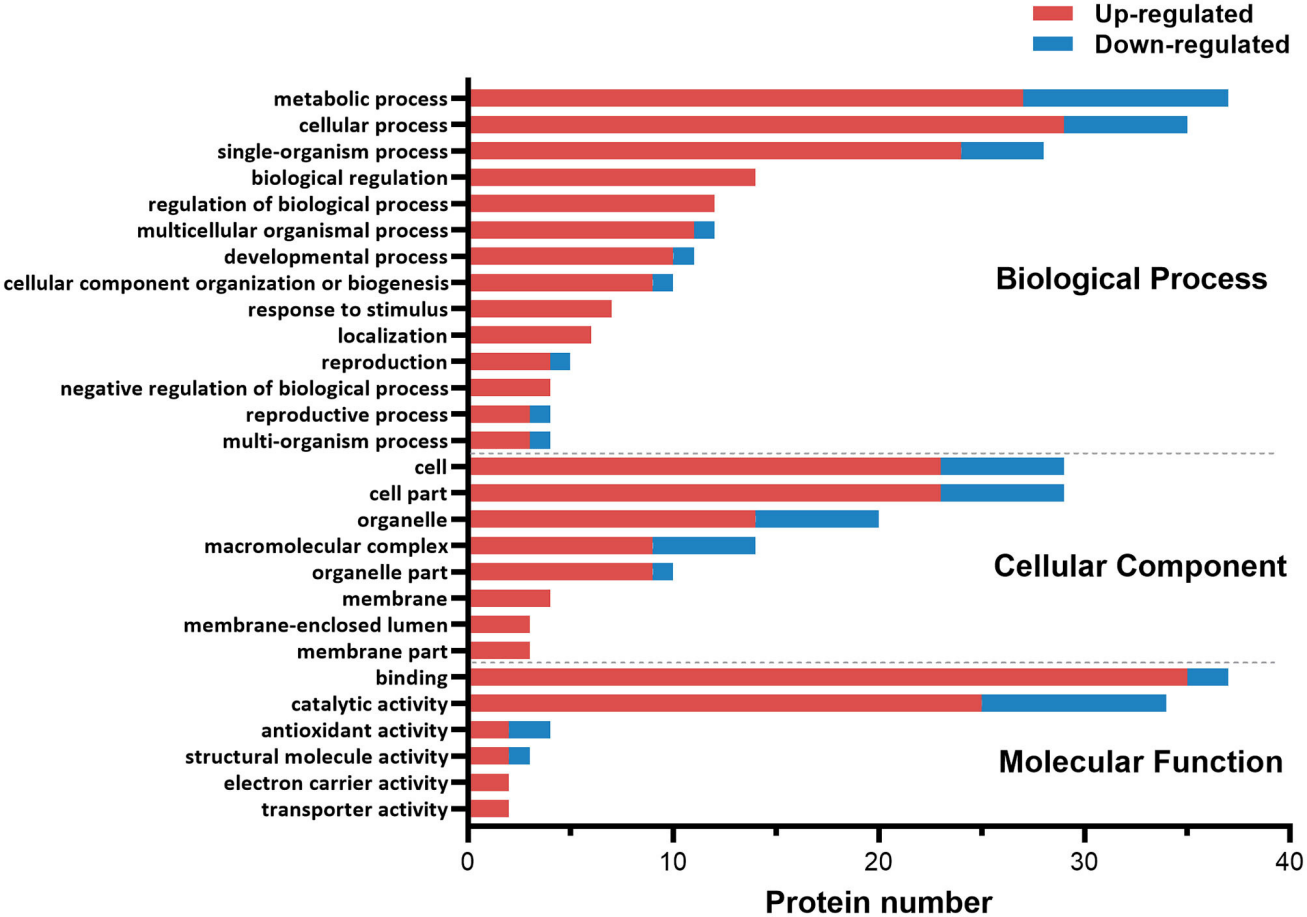
Gene ontology (GO) classification diagram of differentially expressed proteins in the ovary of ESA and ESI crabs. Red and blue represent upregulated and downregulated proteins after ESA, respectively.

The functional category of the DE proteins was analyzed against the Gene Ontology (GO) database using three sets of ontologies: biological process, molecular function, and cellular component (Figure 1). The most abundant proteins in the biological process category were related to metabolic process (GO:0008152, 37 proteins), followed by cellular process (GO:0009987, 35 proteins). In the cellular component category, the proteins associated with cells (GO:0005623, 29 proteins) and cell parts (GO:0044464, 29 proteins) were dominant. In the molecular function category, binding (GO:0005488, 37 proteins) was the most prominent, followed by catalytic activity (GO:0003824, 34 proteins).

### miRNA Expression Modulated by Eyestalk Ablation

To decipher the miRNAs implicated in ovarian maturation induced by ESA, the global ovarian miRNA expression after ESA was investigated using miRNA high-throughput sequencing. Relatively strict criteria were used to identify known and novel miRNAs. Only the small RNAs that aligned to the miRNAs in miRBase with no mismatch were classified as known miRNAs, and only those identified by both miREvo and mirdeep2 software were considered as novel miRNAs. In total, 184 unique miRNAs were identified after eliminating low-abundance miRNAs (reads ≤ 20), among which 100 were novel. Despite stringent criteria used for identifying miRNAs, there is possibility that some of these miRNAs may be degraded products of other RNAs due to lack of reference genome for miRNA prediction.

**Figure 2:**
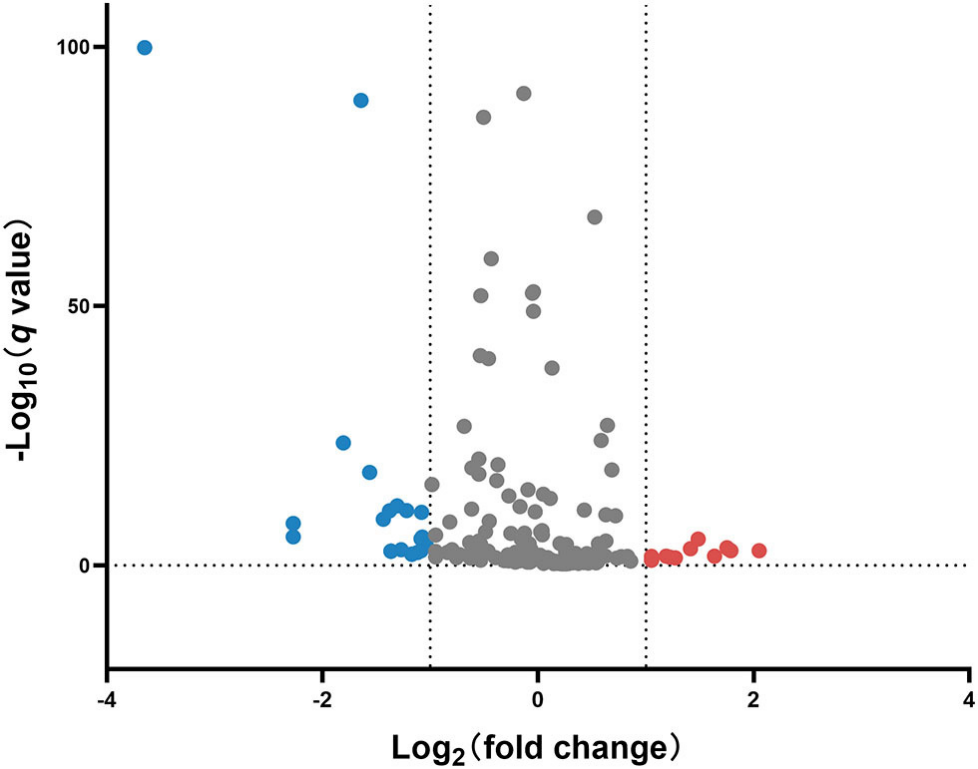
Volcano plot of differentially expressed miRNAs in the ovary of ESA and ESI crabs. Gray, red, and blue dots represent non-significant, upregulated, and downregulated miRNAs after ESA, respectively.

miRNA profile analysis showed that 31 miRNAs exhibited differential expression (under the criteria of fold changes ≥ 2.0 and *p* < 0.05) between ESI and ESA crabs, and most of them (24 miRNAs, 77.42%) were downregulated in ESA individuals (Figure 2 and Table S1).

### *In silico* Correlation of Protein and miRNA and Experimental Validation

**Figure 3:**
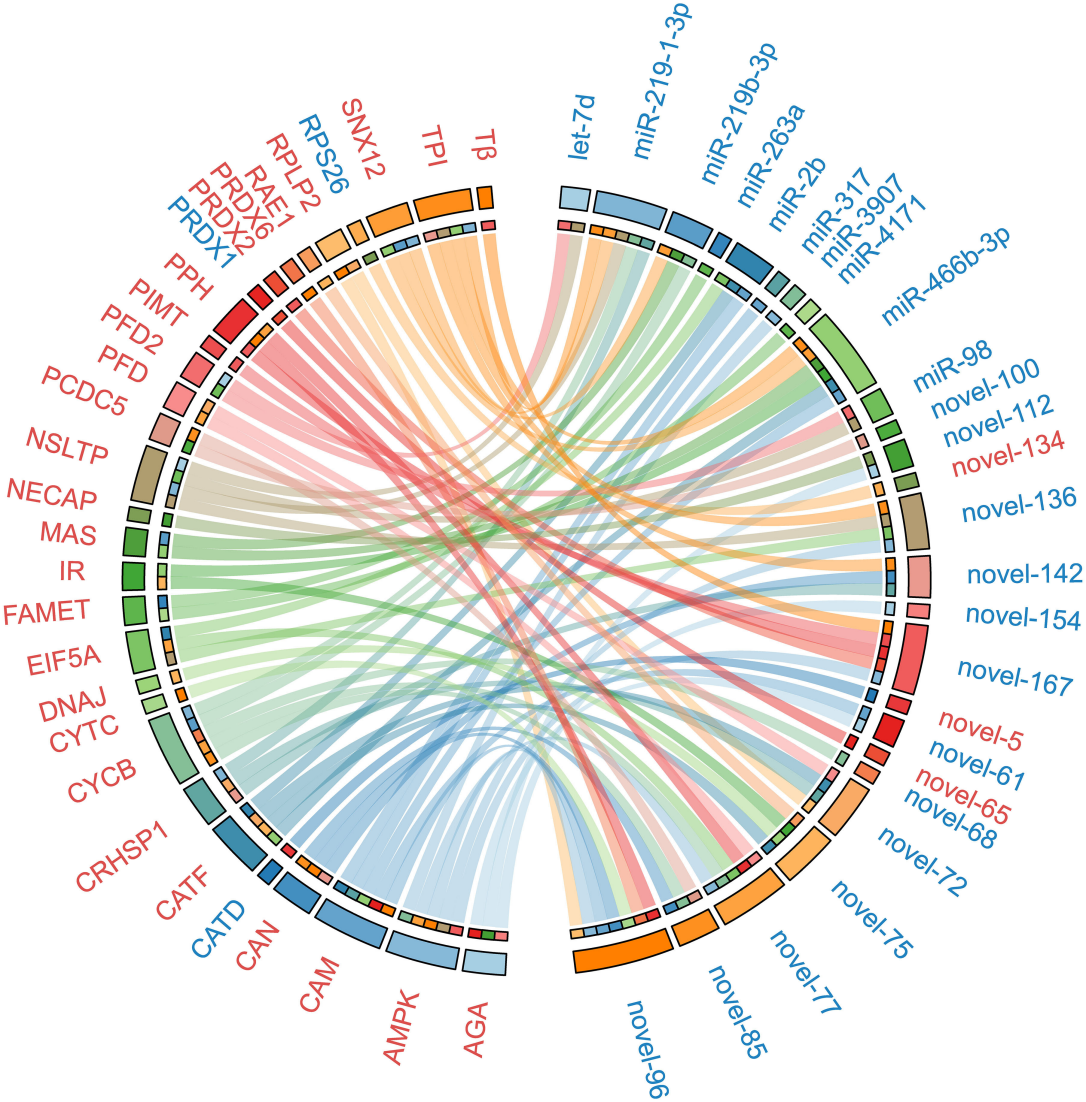
The miRNA-protein regulatory network constructed on the basis of *in silico* miRNA target prediction and inverse correlation of protein and miRNAs expression after ESA. Upregulated miRNAs and proteins are represented in red, and downregulated miRNAs and proteins are represented in blue. The abbreviations in the figure: RPLP2, 60S acidic ribosomal protein P2; NECAP, Adaptin ear-binding coat-associated protein 2; AMPK, AMP-activated protein kinase alpha subunit; AGA, Aspartylglucosaminidase; CRHSP1, Calcium-regulated heat stable protein 1; CAM, Calmodulin; CAN, Calnexin; CATD, Cathepsin D; CATF, Cathepsin F; CATF, Cathepsin F; CYCB, Cyclin B; CYTC, Cytochrome c; DNAJ, DnaJ homolog subfamily B member 9 precursor; EIF5A, Eukaryotic translation initiation factor 5A; FAMET, Farnesoic acid O-methyltransferase; IR, Insulin receptor; MAS, Masquerade-like protein; NSLTP, Non-specific lipid-transfer protein; PRDX1, Peroxiredoxin 1; PRDX2, Peroxiredoxin 2; PRDX6, Peroxiredoxin 6; PPH, Phosphopyruvate hydratase; PFD, Prefoldin; PFD2, Prefoldin subunit 2; PCDC5, Program cell death 5-like; PIMT, Protein-L-isoaspartate O-methyltransferase; RAE1, RAE1 RNA export 1 homolog; RPS26, Ribosomal protein S26; SNX12, Sorting nexin-12; Tβ, Thymosin beta; TPI, Triosephosphate isomerase.

To uncover the potential crosstalk between specific miRNAs and proteins in response to ESA, we constructed a regulatory network based on miRNA target prediction algorithms and inverse correlation of protein and miRNAs expression (Figure 3 and Table 2). The miRNA-protein network was composed of 26 miRNAs and 30 target proteins. Among the targets, several proteins related to methyl farnesoate (MF) synthesis [Farnesoic acid O-methyltransferase (FAMeT)], oocyte meiotic maturation (Cyclin B), calcium signal transduction [Calmodulin (CaM)], and energy metabolism [AMP-activated protein kinase (AMPK), Phosphoglycerate mutase 2 (PGAM2), Triosephosphate isomerase (TPI), Phosphopyruvate hydratase (PPH), and Methylmalonyl-CoA epimerase (MCE)] were identified.

**Table 2:** A list of significantly altered miRNAs and target proteins showing inverse correlation of expression after ESA.

**miRNA**	**miRNA fold change**	**Target protein**	**Target protein fold change**
let-7	0.39	Non-specific lipid-transfer protein	1.54
		Prefoldin subunit 2	1.46
miR-2b	0.41	Calmodulin	1.45
		Cathepsin F	1.28
		Eukaryotic translation initiation factor 5A	1.32
miR-98	0.39	Non-specific lipid-transfer protein	1.54
		Prefoldin subunit 2	1.46
miR-219-1-3p	0.43	Calcium-regulated heat stable protein 1	1.42
		Cyclin B	1.28
		Non-specific lipid-transfer protein	1.54
		Sorting nexin-12	1.70
		Triosephosphate isomerase	1.34
miR-219b-3p	0.40	Cyclin B	1.28
		Masquerade-like protein	1.22
		Sorting nexin-12	1.70
miR-263a	0.47	Farnesoic acid O-methyltransferase	1.67
miR-317	0.21	Calmodulin	1.45
miR-466b-3p	0.41	Calmodulin	1.45
		Cathepsin F	1.28
		Insulin receptor	1.58
		Masquerade-like protein	1.22
		Sorting nexin-12	1.70
		Triosephosphate isomerase	1.34
miR-3907	0.49	AMP-activated protein kinase alpha subunit	1.60
miR-4171	0.39	Farnesoic acid O-methyltransferase	1.67
novel-5	2.69	Cathepsin D	0.79
novel-61	0.29	Aspartylglucosaminidase	1.67
		Calmodulin	1.45
novel-65	2.80	Peroxiredoxin 1	0.78
novel-68	0.34	Cyclin B	1.28
novel-72	0.37	60S acidic ribosomal protein P2	1.39
		Calcium-regulated heat stable protein 1	1.42
		Cathepsin F	1.28
		Prefoldin	1.84
novel-75	0.47	Cathepsin F	1.28
		DnaJ homolog subfamily B member 9 precursor	1.32
		Insulin receptor	1.58
		RAE1 RNA export 1 homolog	1.33
novel-77	0.38	AMP-activated protein kinase alpha subunit	1.60
		Cyclin B	1.28
		Eukaryotic translation initiation factor 5A	1.32
		Phosphopyruvate hydratase	1.32
		Prefoldin	1.84
novel-85	0.21	Calnexin	1.40
		Cyclin B	1.28
		Program cell death 5-like	1.66
novel-96	0.44	60S acidic ribosomal protein P2	1.39
		AMP-activated protein kinase alpha subunit	1.60
		Calnexin	1.40
		Cytochrome c	1.67
		Peroxiredoxin 6	1.61
		Phosphopyruvate hydratase	1.32
		Calmodulin	1.45
novel-100	0.44	Program cell death 5-like	1.66
novel-112	0.32	Aspartylglucosaminidase	1.67
		Similar to adaptin ear-binding coat-associated protein 2	1.25
novel-134	3.45	Ribosomal protein S26	0.78
novel-136	0.46	AMP-activated protein kinase alpha subunit	1.60
		Eukaryotic translation initiation factor 5A	1.32
		Non-specific lipid-transfer protein	1.54
		Triosephosphate isomerase	1.34
novel-142	0.44	Calcium-regulated heat stable protein 1	1.42
		Calnexin	1.40
		Triosephosphate isomerase	1.34
novel-154	0.08	Aspartylglucosaminidase	1.67
novel-167	0.47	AMP-activated protein kinase alpha subunit	1.60
		Peroxiredoxin 2	1.32
		Phosphopyruvate hydratase	1.32
		Protein-L-isoaspartate O-methyltransferase	1.48
		Thymosin beta	1.55

**Figure 4:**
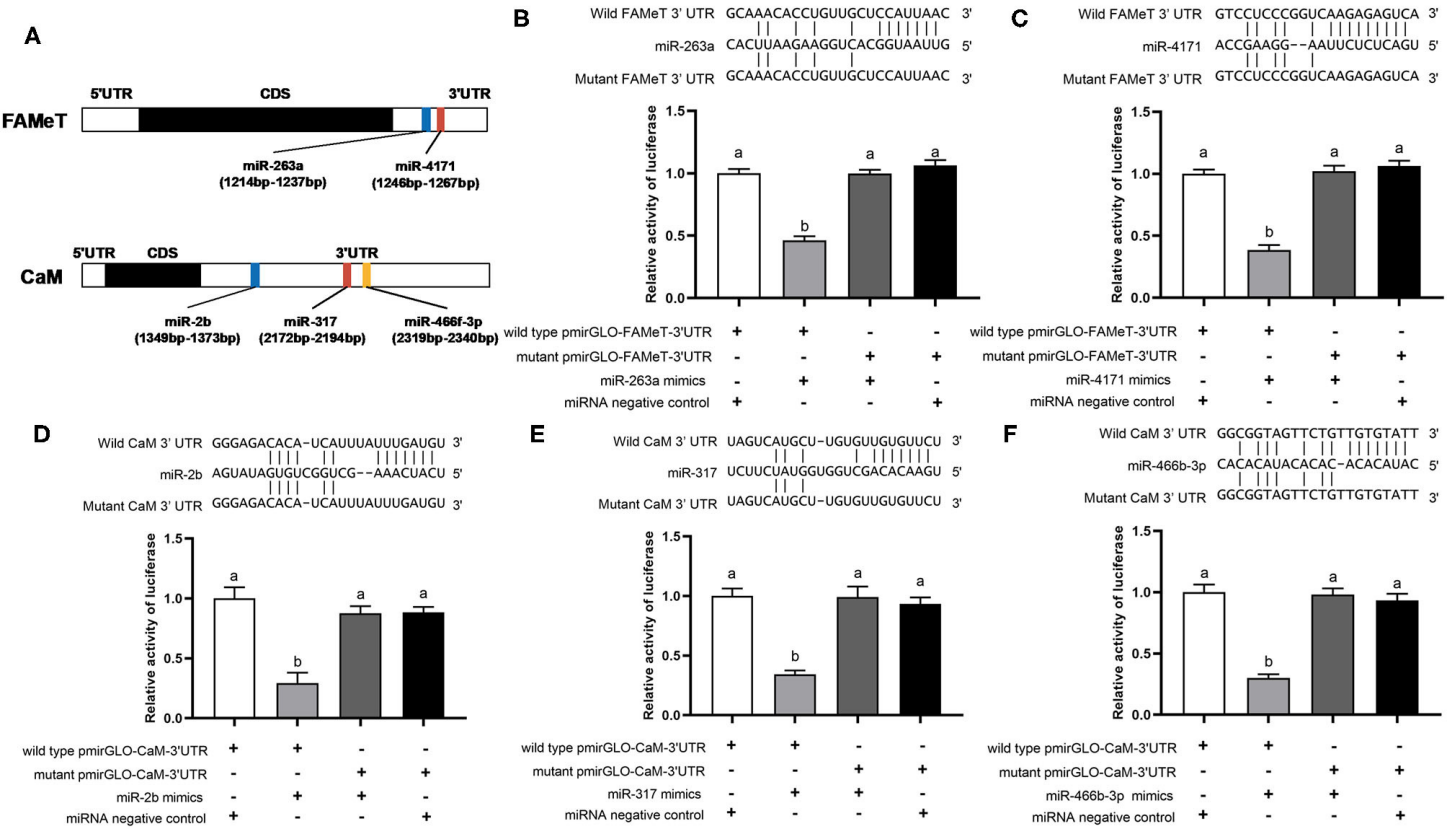
Predicted binding sites of the miRNAs in the 3′ UTRs of their target **(A)** and validation of interactions between the selected pairs of miRNAs and their targets, including miR-263a/FAMeT **(B)**, miR-4171/FAMeT **(C)**, miR-2b/CaM **(D)**, miR-317/CaM **(E)** and miR-466b-3p/CaM **(F)**, via dual luciferase reporter assay. Above the histograms, nucleotide sequences of miRNAs and targets wild-type and mutant 3′ UTR regions are shown. The histograms present relative luciferase activity in HEK293T cells cotransfected with miRNA mimics or miRNA negative control, and pmirGLO vector containing wild-type or mutant 3′ UTR regions of miRNA targets. The relative luciferase activity was calculated by normalizing firefly luciferase activity to Renilla luciferase activity. Different letters on the bars indicate significant differences based on one-way ANOVA followed by Tukey's HSD multiple comparison test (*p* < 0.05).

Using the dual luciferase reporter assay, we validated direct interaction of five miRNA-target pairs (miR-263a/FAMeT, miR-4171/FAMeT, miR-2b/CaM, miR-317/CaM, and miR-466f-3p/CaM) (Figure 4). The results show that the relative luciferase activity (firefly luciferase activity/Renilla luciferase activity) was significantly reduced after pmirGLO-FAMeT-3′UTR was cotransfected with miR-263a or miR-4171 and pmirGLO-CaM-3′UTR was cotransfected with miR-2b, miR-317, or miR-466f-3p, which indicates the interaction between the miRNAs and their targets.

**Figure 5:**
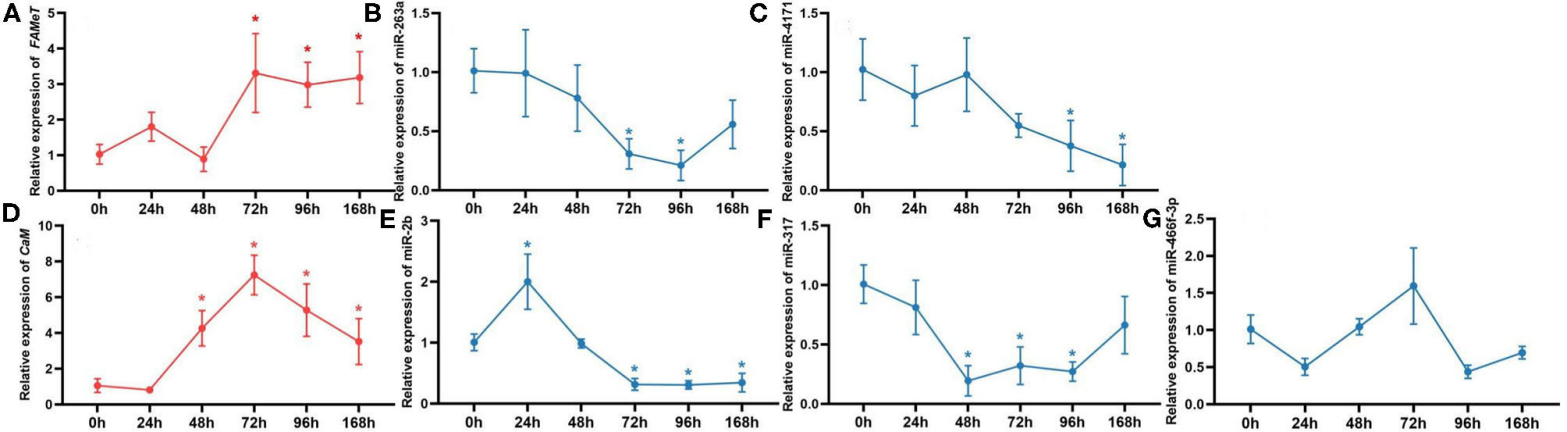
The relative expression of *FAMeT* **(A)** and *CaM* **(D)** and their corresponding miRNAs, including miR-263a **(B)**, miR-4171 **(C)**, miR-2b **(E)**, miR-317 **(F)**, and miR-466f-3p **(G)** at 0, 24, 48, 72, 96, and 168 h after ESA. The asterisk indicates that the expression significantly differs from that at 0 h (*p* < 0.05).

To further validate the correlation between the miRNAs and their targets and investigate their temporal express pattern after ESA, we analyzed the levels of the miRNAs and their target genes at different times following ESA. The results show that expression of miR-263a, miR-4171, miR-2b, and miR-317 negatively correlates with that of their target genes (Figure 5) (miR-263a/*FAMeT*: *p* < 0.01; miR-4171/*FAMeT*: *p* < 0.01; miR-2b/*CaM*: *p* < 0.01; miR-317/*CaM*: *p* < 0.01), and there is no significant correlation between expression levels of miR-466f-3p and *CaM* (*p* > 0.05). *FAMeT* increased significantly from 72 h and maintained high expression until 168 h after ESA, whereas miR-263a and miR-4171 exhibited lower expression at those time points. *CaM* showed a significant upregulation from 48 to 168 h, and miR-2b and miR-317 were downregulated during these time periods. These results further confirm that miR-263a, miR-4171, miR-2b, and miR-317 are involved in regulating the expression of FAMeT and CaM after ESA.

## Discussion

Despite the undesirable effects, eyestalk ablation is currently the most effective method to induce ovarian maturation in the commercial hatchery of decapods. To date, the molecular mechanisms underlying the induced maturation have not been fully understood, which hinders development of new techniques for maturation manipulation. Previously, many studies have been focused on transcriptional regulation in response to ESA (35–37). Considering the fact that proteins and miRNAs form the cellular end point (proteins) of phenotypic features and their regulatory elements (miRNA), the present study concentrated on changes that occur in protein and miRNA expression. The results here present for the first time extensive ovarian proteome and miRNA profiling reflecting the alterations induced by ESA. Proteomic analysis using iTRAQ shows that 163 proteins are differentially expressed in the ovary of intact and ablated crabs, and among them, proteins regulating vitellogenesis, oocyte meiosis, and energy metabolism are identified, indicating their involvement in promoting ovarian maturation after ESA. miRNA transcriptome results show that 31 miRNAs exhibit differential expression after ESA. By integrating bioinformatic algorithms with expression data of miRNA and protein, we identify 30 targets of the DE miRNAs and define the potential roles of miRNA in maturation induced by the ablation.

### Vitellogenesis

Vitellogenin (Vg), the precursor of the major egg yolk proteins, is regarded as a reliable marker of ovarian maturation in decapods as its expression is positively correlated to ovarian maturation levels (11). Consistent with the results of Vg transcript levels in previous studies (38, 39), Vg protein level in the ovary was enhanced significantly after ESA in this study, confirming that ESA leads to increased ovarian vitellogenesis in the swimming crab. In crustaceans, various Vg synthesis sites have been reported in different species. For *P. trituberculatus*, vitellogenesis is demonstrated to occur both within the oocyte (endogenous vitellogenesis) and external to the oocyte (exogenous vitellogenesis), and exogenous Vg is the major source of Vg, which is synthesized in hepatopancreas, transported via hemolymph, and absorbed by oocytes or follicle cells through receptor-mediated endocytosis (40). In our study, an elevated level of Vg receptor (VgR) was observed in the ovary of the ablated crabs compared with that of the intact crabs. This result suggested that ESA can promote accumulation of exogenous vitellogenesis by upregulating VgR expression and facilitating uptake of Vg from hemolymph.

### Methyl Farnesoate Metabolism

Methyl farnesoate (MF), a sesquiterpene compound, plays a crucial role in reproduction regulation of decapods (41). Previous studies demonstrate that there is a positive correlation between MF titer and ovarian maturation in decapods, and injection of MF stimulates oocyte growth and vitellogenesis (1, 42, 43). The biosynthetic pathway of MF is similar to the general mevalonate pathway for acyclic isoprenoids, and the final step is catalyzed by FAMeT (44–46). In this study, a significant upregulation of FAMeT in ovary was observed after ESA, indicating that ESA resulted in an increased rate of MF synthesis. This result is in accordance with previous studies that find the MF levels are negatively regulated by eyestalk neuropeptides, and removal of the eyestalk leads to elevated levels of MF (46). MF level is controlled by both anabolism and catabolism. Interestingly, a key enzyme in MF degradation, namely juvenile hormone esterase-like carboxylesterase (JHE-like CXE), was also found upregulated in ESA crabs, indicating an increase in MF catabolism in addition to MF anabolism. It is reported that a high level of MF has a deleterious effect on oocyte growth and development, resulting in oocyte degeneration (47). The increased MF catabolism in the ovary after ESA may represent a protective mechanism to regulate MF levels, avoiding the detrimental effects of excessive MF.

### Calcium Signals

Calcium signals are demonstrated to be essential for oocyte maturation in mammals, fish, mollusks, and crustaceans (48–50). Elevated concentration of intracellular calcium in the oocyte can activate CaM, a molecular switch to regulate the network of calcium signaling and, in turn, trigger a cascade of downstream signaling, leading to activation of maturation-promoting factor (MPF), which is a heterodimeric complex composed of Cell division cycle 2 (Cdc 2) and Cyclin B and responsible for triggering G2/M-phase transition in oocytes (51–53). It is reported that ESA results in an increased level of CaM mRNA in the ovary of the red swamp crayfish *Procambarus clarkia*, and knockdown of CaM expression by RNA interference suppresses the ovarian maturation induced by ablation (54). In this study, CaM, as well as Cdc 2 and Cyclin B, were upregulated after ESA. These results together with previous findings suggest that ESA can stimulate meiotic maturation through calcium signaling. In mammals, GnRH signaling can trigger calcium signaling, thereby inducing oocyte maturation. Similarly, a recent study in the black tiger prawn *Penaeus monodon* finds that genes in the GnRH pathway exhibit an early induction pattern after ESA and speculates that GnRH signaling leads to the activation of calcium signaling (11). However, upregulation of the proteins in the GnRH signaling pathway was not observed in this study. It is possible that activation of GnRH signaling is transient at the onset of oocyte maturation after ESA. Further study is required to confirm the upstream signals triggering calcium-signaling pathways after ESA.

### Metabolism

KEGG analysis shows that the proteins in metabolic pathways account for the largest proportion of DE proteins (22.70%), and they are mapped to a wide range of pathways related to metabolism, such as carbohydrate, amino acid, energy, lipid, and nucleotide metabolism, indicating that ESA has an extensive impact on the metabolism in the *P. trituberculatus* ovary. It is noteworthy that a significant upregulation of AMPK was observed after ESA. As a key regulator that maintains cellular energy homeostasis, AMPK reprograms cellular metabolism from anabolism to catabolism by controlling lipid and glucose metabolism when activated by falling energy status (55, 56). In accordance with AMPK upregulation, several important enzymes in glycolysis (PGAM2, TPI, and PPH), tricarboxylic acid cycle (ACO), and fatty acid catabolism (MCE) were also found upregulated following ablation. Previous studies in other decapods show that ESA can promote the transportation of glucose and lipid from the hepatopancreas to the ovary (57, 58). Those findings and our results together indicate ESA results in an increased energy requirement for the accelerated ovarian maturation. Hence, in aquaculture practice, the feeds for the broodstocks should be adjusted to meet the high energy demand after ablation.

### The Roles of miRNA

Accumulating evidence suggests that miRNAs are critical in ovarian development of crustacean (59, 60). However, their functions in ESA-induced ovarian maturation are still unclear. In this study, 31 miRNAs were differentially expressed in ESA and ESI crabs. To uncover the functions of these miRNA during accelerated ovarian maturation induced by ESA, we integrated miRNA transcriptome with proteome data sets. Based on *in silico* miRNA target prediction and inverse correlation of protein and miRNA expression, we generated a network encompassing 26 miRNAs, 30 target proteins, and 71 potential functional associations (Figure 3). Among the targets, a number of proteins mentioned above, including FAMeT, Cyclin B, CaM, AMPK, TPI, and PPH, were identified, suggesting that miRNAs may play important roles in ESA-mediating ovarian maturation through regulating proteins associated with MF synthesis, calcium signals, and energy metabolism. Within the miRNA-protein network, most miRNAs have multiple targets, and inversely, many proteins are targeted by several miRNAs, implying a complex post-transcriptional regulation between miRNAs and proteins in response to ESA. To experimentally validate the synergistic target regulation of the miRNAs, we tested FAMeT regulation by miR-263a and miR-4171, and CaM regulation by miR-2b, miR-317, and miR-466f-3p, using the dual luciferase reporter assay. The results indicate that both miR-263a and miR-4171 directly regulate FAMeT expression, and miR-2b, miR-317, and miR-466f-3p repress expression of CaM. In addition, we investigated the expression pattern of the miRNAs and their target genes after ESA, and the results show significant negative correlation between miR-263a, miR-4171, and *FAMeT* and between miR-2b, miR-317, and *CaM*. Taken together, these data demonstrate that multiple miRNAs could function cooperatively to regulate FAMeT and CaM expression, and thereby participate in inducing ovarian maturation after ESA.

## Conclusions

In summary, we characterize the changes in ovarian proteome and miRNA transcriptome in *P. trituberculatus* after ESA. The results suggest that alterations in MF metabolism and calcium signaling are crucial for promoting ovarian maturation, and ESA results in a higher energy production to meet the increased energy demand in the ovary. Furthermore, our study reveals a miRNA-mediated mechanism for inducing ovarian maturation. These findings improve our understanding of the molecular mechanisms of ESA-induced ovarian maturation and lay the foundation for developing an alternative technique for maturation manipulation without ESA in decapod crustaceans.

## Materials and Methods

### Animal Collection and Eyestalk Ablation

All the experimental procedures involving the handling and treatment of the crabs used in this study were approved by the Institutional Animal Care and Use Committee of Yellow Sea Fisheries Research Institute prior to initiation of experiments. Female *P. trituberculatus* at 6-month age (198.65 ± 23.77 g) were collected from Haifeng Company, Weifang, China. The crabs were acclimated at laboratory conditions (temperature, 21°-23°C; salinity, 30–31) for 3 weeks. Then, three crabs were bilaterally eyestalk-ablated, and wounds were cauterized to minimize loss of hemolymph. Based on the results of our preliminary experiment, 96 h post ESA was selected as sample time; three eyestalk-ablated individuals (ESA) and three individuals with intact eyestalks (ESI) were placed in an ice bath until anesthetized, and the ovaries were collected and immediately frozen in liquid nitrogen and stored at −80°C.

During the period of the experiment, all the crabs were fed daily at 17:00 with live Manila clam *Ruditapes philippinarum*, and the feces and leftover feed were removed prior to feeding. Aeration was provided continuously, and the photoperiod was 12 h light:12 h dark. Seawater was filtered using a sand filter, and one third to one half of the rearing water was exchanged using fresh equi-temperature seawater. Water pH was around 7.5, and ammonia was <0.23 mg L^−1^. Water salinity, pH, and ammonia were determined with a salinity refractometer (AIAGO, Japan), pH meter (WTW, Germany), and Hypobromite methods, respectively.

### Protein Extraction, Digestion, and iTRAQ Labeling

iTRAQ analysis was performed at Beijing Genomics Institute (BGI, Shenzhen, China). The ovary of each crab from the ESI and ESA groups were disrupted in lysis buffer with enzyme inhibitors by TissueLyser (Qiagen, USA). The mixtures were centrifuged at 25,000 g for 20 min, and the supernatant was carefully removed and mixed with 5 volume of cold acetone and stored at −20°C for 2 h prior to centrifuging. The pellets were dissolved with lysis buffer, and 10 mM dithiothreitol (DTT) was added and maintained at 56°C for 1 h to reduce the disulfide bond of peptides. Then, 55 mM IAM was added to the solution and kept in a dark room for 45 min. After adding 5 volume of chilled acetone into the solution and kept at −20°C for 2 h, the solution was centrifuged again, and the pellet was dissolved with lysis buffer to get a protein solution. The protein concentration was determined using the Bradford method.

The protein solutions (100 μg) from each sample were digested with Trypsin gold (Promega, USA). After digestion, the peptides were vacuum centrifuged to dryness and dissolved with 0.5 M TEAB. The iTRAQ labeling of peptides was performed on the ESI and ESA groups using the iTRAQ Reagent 8-plex kit (Applied Biosystems, USA) according to the manufacturer's protocol.

### Protein Identification, Quantification, and Functional Analysis

Raw LC-MS/MS data was converted into MGF format with the exported ProteoWizard tool (61). Proteins were identified with Mascot version 2.3.02 (Matrix Science, UK) against *P. trituberculatus* ovarian transcriptome. The proteins containing at least one unique set of spectra were used for the following quantification analysis with IQuant software (62). The proteomic data set was deposited in the iProX database under the accession number IPX0002268000. A *q* value <= 0.05 and foldchange >= 1.2 were set as the threshold for differentially expressed proteins (DEPs). The GO and KEGG databases were used to classify and group the DEPs.

### RNA Extraction, Library Construction, and miRNA Sequencing

Total RNA was extracted from the ovary of the same crabs used for proteomic analysis with TRIzol reagent (Invitrogen, USA) and purified with a mirVana miRNA Isolation Kit following the manufacturer's protocol (Ambion, USA). Total RNA degradation and contamination was accessed on 1% agarose gels. Quantity and integrity of the RNA samples were determined using a Nano Photometer spectrophotometer (Implen, USA) and Bioanalyzer 2100 system (Agilent Technologies, USA). The RNA concentration was measured using the Qubit RNA Assay Kit in the Qubit 2.0 Flurometer (Life Technologies, USA).

For the ESI or ESA small RNA library, an equal amount of RNA samples from each replicate were pooled together. The sequencing libraries were generated using NEBNext Multiplex Small RNA Library Prep Set for Illumina (New England Biolabs, USA) according to the manufacturer's protocol, and index codes were added to attribute sequences to each sample. Library quality was assessed on the Agilent Bioanalyzer 2100 system (Agilent Technologies, USA) using DNA High Sensitivity Chips. The clustering of the index-coded samples was performed on a cBot Cluster Generation System using TruSeq SR Cluster Kit v3-cBot-HS (Illumina, USA) according to the manufacturer's instructions. After cluster generation, the small RNA libraries were sequenced on an Illumina Hiseq 2500 platform at Novogene Company, Beijing, China. The small RNA sequencing data set was deposited in NCBI Sequence Read Archive (SRA) under the accession numbers PRJNA639350.

### miRNA Data Analysis

After Illumina sequencing, clean reads were obtained by removing low-quality sequences, adapter-contaminated tags, and reads with poly N (where N represents unknown base). All the clean reads were searched against GenBank and Rfam databases to exclude known non-coding RNAs, including rRNAs, tRNAs, snRNAs, and snoRNAs. Any reads encoding proteins were also removed by blasting against the reference unigenes derived from the gonadal transcriptome data set of *P. trituberculatus*. Then, the remaining sequences were searched against the miRNAs from all the animals in miRBase to identify known miRNAs. Only the sequences that aligned to the miRNAs in miRBase without any mismatch were considered as known miRNAs. To predict novel miRNAs, the remainder unannotated small RNA sequences were analyzed with an integrated combination of miREvo (63) and mirdeep2 (64). The software identified novel miRNAs by exploring the secondary structure, the Dicer cleavage site, and the minimum free energy of the small RNA tags unannotated in the former steps, and the threshold score set as ≥ 50. The miRNAs predicted by the software were considered as novel miRNAs.

To analyze the expression profiles of the miRNAs in the ovary of ESA and ESI crabs, the read counts of miRNAs were normalized into TPM (transcript per million) through the normalization formula: Normalized expression = (Mapped reads/Total reads) × 106 (65). Differential expression analysis of the two libraries was performed using the DEGseq R package (65). *P* values were adjusted using *q* value (66). The criteria of *q* < 0.01 and |log2(fold-change)| > 1 was set as the threshold for defining statistically different expression. Because genome information for the swimming crab is not available, 3′UTR sequences extracted from the *P. trituberculatus* gonad transcriptome data were used to predict putative targets of the identified miRNAs with miRanda software (67).

### miRNA Target Validation

Five selected miRNA-target pairs (miR-263a/FAMeT, miR-4171/FAMeT, miR-2b/CaM, miR-317/CaM, and miR-466f-3p/CaM) were validated with dual luciferase reporter assay. The 3′ UTR sequences of FAMeT and CaM containing wild-type and mutant miRNA binding sites were artificially synthesized by General Biol Co. Ltd. (Hefei, China) and cloned into pmirGLO dual-luciferase reporter vector (Promega, USA) using *Sac*I and *Sal*I restriction sites. The plasmids were transformed into TOP10 *E. coli* cells and purified with a TIANprep Mini Plasmid Kit (Tiangen, China), and all the insertions were certified by DNA sequencing. The synthesized 3′ UTR sequences of FAMeT and CaM are shown in the supplementary materials (Table S2).

For the luciferase reporter assay, HEK293T cells were seeded in 24-well plates and transfected with 50 nM miRNA mimics or scrambled miRNA, and 1 μg luciferase reporter plasmid pmirGLO-wild type or pmirGLO-Mutant using Exfect 2000 Transfection Reagent (Vazyme, China). At 48 h after transfection, firefly and Renilla luciferase activities were determined using the dual luciferase reporter assay system (Promega, USA). The firefly luciferase signal was normalized to the Renilla luciferase signal. All experiments were performed in three replicates. The normalized firefly luciferase activity was compared between different groups using Tukey's HSD multiple comparison test (*p* < 0.05).

To further validate the expression correlation between the five selected miRNA target pairs, we analyzed levels of the miRNAs and their target at different time (0, 24, 48, 72, 96, and 168 h) after ESA. Because there is no proper antibody available, we measured mRNA levels of the targets instead. Total RNA of the samples was extracted using the RNAprep Pure Kit (Tiangen, China), and reverse transcription and RT-PCR of the target genes were performed with the FastKing RT Super Mix (Tiangen, China) and SuperReal PreMix Plus Kit (Tiangen, China), respectively, according to the manufacturer's protocol. The RT-PCR was programmed at 95°C for 15 min, followed by 40 cycles of 95°C for 10 s, 58°C for 20 s, and 72°C for 30 s. miRNA was isolated using the miRcute miRNA Isolation Kit (Tiangen, China) following the manufacturer's instruction, and reverse transcription and RT-PCR of the miRNAs were performed using miRcute miRNA First-strand cDNA Synthesis Kit (Tiangen, China) and miRcute Plus miRNA qPCR Kit (Tiangen, China). The RT-PCR was programmed at 95°C for 15 min, followed by 40 cycles of 94°C for 20 s and 60°C for 34 s. Three biological replicates were measured at each time point, and each measurement was performed in triplicate. Relative levels of miRNAs and mRNAs were normalized to the U6 snRNA and β-actin, respectively, in each sample using the comparative C_T_ method (68). All the primers are shown in Supplementary Materials (Table S3). The correlation in expression between the miRNAs and their targets was determined using Spearman's correlation analysis.

## Data Availability Statement

The small RNA sequencing dataset was deposited in NCBI sequence read archive (SRA) under the accession numbers PRJNA639350.

## Ethics Statement

The animal study was reviewed and approved by the Institutional Animal Care and Use Committee of Yellow Sea Fisheries Research Institute.

## Author Contributions

XM and PL: conceptualization, resources, supervision, project administration, and funding acquisition. MZ, XM and PL: methodology and investigation. XM, MZ, and JL: software, validation, formal analysis, data curation, and original draft. XM, MZ, and PL: writing, review, and editing.

## Conflict of Interest

The authors declare that the research was conducted in the absence of any commercial or financial relationships that could be construed as a potential conflict of interest.
