# Supplementary material for: Integrative Proteomic and MicroRNA Analysis: Insights Into Mechanisms of Eyestalk Ablation-Induced Ovarian Maturation in the Swimming Crab Portunus trituberculatus
Source: Front Endocrinol (Lausanne). 2020 Aug 14;11:533. doi: 10.3389/fendo.2020.00533 (PMC7456853; doi:10.3389/fendo.2020.00533)
Supplement: Supplementary file 1 [file Table_1.DOCX]

**Table S1. The miRNAs which exhibited differentially expression after ESA**

| **miRNA** | **Sequences (5'-3')** | **log2 Fold change** |
| --- | --- | --- |
| novel-159 | UAUUAGGCAAAAUAUUGAGGAAGUG | 2.05 |
| novel-134 | UGUUUACUGCUGGCUGUGAUACAUC | 1.79 |
| miR-184 | UGGACGGAGAACUGAUAAGGG | 1.76 |
| novel-115 | CACCAGAGAAACUGUGAGGACGAGU | 1.75 |
| novel-65 | UCACUCACUGGUGACGCUCUGGG | 1.48 |
| novel-5 | GCUGUUGAGACUCUCCUGGACGAGU | 1.43 |
| miR-184-3p | UGGACGGAGAACUGAUAAGGGU | 1.41 |
| miR-3907 | AGGUGCUCCAGGCUGGCUCACA | -1.04 |
| novel-167 | UUUCAUGAUCCAGUCUGUAAAUCUG | -1.07 |
| novel-75 | UAGUAUAAUCGCCAACAAGCUGAGU | -1.08 |
| miR-263a | GUUAAUGGCACUGGAAGAAUUCAC | -1.09 |
| novel-136 | UAUUUGUCGUUUGCUCUCCAUUAUU | -1.12 |
| novel-100 | UCGAAUCAUUAGAAUGUUGGAAAGU | -1.17 |
| novel-142 | UUGGUUCUUAGGGUAUGAGCGCGUA | -1.17 |
| novel-96 | UCAAAUAUUAGGGUGCGUCUUCC | -1.17 |
| miR-219-1-3p | AAGAAUUGUGUCUGGACAUCGG | -1.22 |
| miR-466f-3p | CAUACACACACACAUACACAC | -1.27 |
| miR-2b | UCAUCAAAGCUGGCUGUGAUAUGA | -1.28 |
| miR-219b-3p | AGAAUUGUAUCUGGACAUCUGU | -1.31 |
| let-7 | UGAGGUAGUAGGUUGUAUAGUU | -1.36 |
| miR-4171 | UGACUCUCUUAAGGAAGCCA | -1.36 |
| miR-98 | UGAGGUAGUAAGUUGUAUUGU | -1.36 |
| novel-60 | UAAAGUCGCUGGGGCUUGACCC | -1.36 |
| novel-77 | UUAUGAUGUUCAGUAGUGUAGCACU | -1.38 |
| novel-72 | UACAUUCACCUGUAUCUGGGUUCUG | -1.43 |
| novel-68 | UAGGAUCUGUCUUCAUCUGAAUUUC | -1.56 |
| novel-112 | UAGCUAGUGUCUGUACGACAACGUG | -1.64 |
| novel-61 | AAAGAAGACAUCGGACACACUCCUU | -1.81 |
| novel-85 | AGGGGUGUGCAACAAAUUAUUUUU | -2.27 |
| miR-317 | UGAACACAGCUGGUGGUAUCUUCU | -2.28 |
| novel-154 | AGAGACCGUACUGAGGCUCACGUGU | -3.65 |
