# Supplementary material for: Integrative Proteomic and MicroRNA Analysis: Insights Into Mechanisms of Eyestalk Ablation-Induced Ovarian Maturation in the Swimming Crab Portunus trituberculatus
Source: Front Endocrinol (Lausanne). 2020 Aug 14;11:533. doi: 10.3389/fendo.2020.00533 (PMC7456853; doi:10.3389/fendo.2020.00533)
Supplement: Supplementary file 2 [file Table_2.DOCX]

| **Table S2. The 3′ UTR sequences synthesized for pmirGLO vector construction*** | | |
| --- | --- | --- |
| **Gene name** | **Corresponding miRNA** | **Sequence (5′-3′)** |
| FAMeT-  wild | miR-263a  miR-4171 | GCCGCCATGTTGTCCGCCACCTATGCTACGTAATACAGCTTACATCCTTGTCTTCGTTTTGAGTTTATAATAAAATTTCCGAAGCCAGAGAAGCACTGCGAAGGTGAAGCCTCGTGCAAACACCTGTTGCTCCATTAACCTTCACATGTCCTCCCGGTCAAGAGAGTCAAGAGGAAGGGGTATTTTTCATACTTTGTAGCTTCACGTCATT |
| FAMeT-  mutant | miR-263a  miR-4171 | GCCGCCATGTTGTCCGCCACCTATGCTACGTAATACAGCTTACATCCTTGTCTTCGTTTTGAGTTTATAATAAAATTTCCGAAGCCAGAGAAGCACTGCGAAGGTGAAGCCTCGTGCAAACACCTGTTGCTGGTAATTCCTTCACATGTCCTCCCGGTCAACTCTCAGAAGAGGAAGGGGTATTTTTCATACTTTGTAGCTTCACGTCATT |
| CaM-  wild-1 | miR-2b | TGCCAAGGTTGGTGTGCTGGGGTTGCTGCTCGAGGGAGTAACCAGCTTTACACTCCGCTGCTACCAGTATTTGCACTTCCTGGGAGACACATCATTTATTTGATGTGTCAAGTGTAGCAGGAGGGCCAGGCCTGTTCCTGGTACCTCACCCTAACACCTTGGGGGTCTGCCACCCTGCCAGTCTCAACATCACCGAGC |
| CaM-  mutant-1 | miR-2b | TGCCAAGGTTGGTGTGCTGGGGTTGCTGCTCGAGGGAGTAACCAGCTTTACACTCCGCTGCTACCAGTATTTGCACTTCCTGGGAGACACATCATTTAAAACTACTGTCAAGTGTAGCAGGAGGGCCAGGCCTGTTCCTGGTACCTCACCCTAACACCTTGGGGGTCTGCCACCCTGCCAGTCTCAACATCACCGAGC |
| CaM-  wild-2 | miR-317  miR-466f-3p | CACTTAGAATATTATTTTCCTCATTCATTTTAGCCAATATGGCATTGTTGGTTTTTTGAAGCTTATCTGGGTCTCATCAGTTGACGGAAGGTGTGTAGTCATGCTTGTGTTGTGTTCTCAGCACCCAGGGTGGCCAAATCCTGCCCATCCCGGCTCAAGCTTGGGATTGTAGGTAGGACATGGTATCTTCCCTGCAGGCTATTTTAGATTCATTTGGGGGAACCTTTTTATTGGTGGTGCGTGGCGGTAGTTCTGTTGTGTATTTGTATCAACAATGTGACACGCGACCAAACTCCGCTTCGCGATGAACATTCCTTTATGCTGCTATGTTTGTGAGGGGCGTGGCACGTTCAGATGGCTGCCTCGCCCATCTTGTTTTAGTG |
| CaM-  mutant-2 | miR-317  miR-466f-3p | CACTTAGAATATTATTTTCCTCATTCATTTTAGCCAATATGGCATTGTTGGTTTTTTGAAGCTTATCTGGGTCTCATCAGTTGACGGAAGGTGTGTAGTCATGCTTGTGTACACAAGTCAGCACCCAGGGTGGCCAAATCCTGCCCATCCCGGCTCAAGCTTGGGATTGTAGGTAGGACATGGTATCTTCCCTGCAGGCTATTTTAGATTCATTTGGGGGAACCTTTTTATTGGTGGTGCGTGGCGGTAGTTCTGTACACATATTGTATCAACAATGTGACACGCGACCAAACTCCGCTTCGCGATGAACATTCCTTTATGCTGCTATGTTTGTGAGGGGCGTGGCACGTTCAGATGGCTGCCTCGCCCATCTTGTTTTAGTG |

*the sequences which has the same color with the miRNAs represents the binding sites of the miRNAs
