# Supplementary material for: Integrative Proteomic and MicroRNA Analysis: Insights Into Mechanisms of Eyestalk Ablation-Induced Ovarian Maturation in the Swimming Crab Portunus trituberculatus
Source: Front Endocrinol (Lausanne). 2020 Aug 14;11:533. doi: 10.3389/fendo.2020.00533 (PMC7456853; doi:10.3389/fendo.2020.00533)
Supplement: Supplementary file 3 [file Table_3.DOCX]

**Table S3. Primers used in qRT-PCR Assay**

| Primer name | Primer Sequence |
| --- | --- |
| miR-2b | CGCTCATCAAAGCTGGCTGTGAT |
| mir-263a | TCGTTAATGGCACTGGAAGAATTCAC |
| mir-317 | CCGTGAACACAGCTGGTGGTATCT |
| mir-466f-3p | GCGCATACACACACACATACACAC |
| mir-4171 | CGGCTGACTCTCTTAAGGAAGCCA |
| U6 Forward | CATATACTAAAATTGGAACGATACAG |
| U6 Reverse | AACGCTTCACGATTTTGCGT |
| FAMeT Forward  FAMeT Reverse | TTCGCTTCCAGGTCAAGACG  CCAGAACTCGCGATACTCCG |
| CaM Forward  CaM Forward | ACGGCAACGGCTTCATCTCT  AAGGGAGTTCAGACGCCGAG |
| β-actin Forward | AGCGAGGCTACACCTTCAC |
| β-actin Reverse | TCCAGGGAGGAGGAAGAAG |
